# Pan-cancer analysis reveals PDK family as potential indicators related to prognosis and immune infiltration

**DOI:** 10.1038/s41598-024-55455-1

**Published:** 2024-03-07

**Authors:** Shigui Tao, Kunlin Tao, Xiaoyong Cai

**Affiliations:** 1 The Second Affiliated Hospital of Guangxi Medical University, Nanning, China; 2 Guiping People’s Hospital, Guangxi, China

**Keywords:** PDK family, Pan-cancer, Survival analysis, Immune infiltration, Therapeutic target, Cancer, Immunology

## Abstract

Pyruvate dehydrogenase kinases (PDKs) play a key role in glucose metabolism by exerting negative regulation over pyruvate dehyrogenase complex (PDC) activity through phosphorylation. Inhibition of PDKs holds the potential to enhance PDC activity, prompting cells to adopt a more aerobic metabolic profile. Consequently, PDKs emerge as promising targets for condition rooted in metabolic dysregulation, including malignance and diabetes. However, a comprehensive exploration of the distinct contribution of various PDK family members, particularly PDK3, across diverse tumor types remain incomplete. This study undertakes a systematic investigation of PDK family expression patterns, forging association with clinical parameters, using data from the TCGA and GTEx datasets. Survival analysis of PDKs is executed through both Kaplan–Meier analysis and COX regression analysis. Furthermore, the extent of immune infiltration is assessed by leveraging the CIBERSORT algorithm. Our study uncovers pronounced genetic heterogeneity among PDK family members, coupled with discernible clinical characteristic. Significantly, the study establishes the potential utility of PDK family genes as prognostic indicators and as predictors of therapeutic response. Additionally, our study sheds light on the immune infiltration profile of PDK family. The results showed the intimate involvement of these genes in immune-related metrics, including immune scoring, immune subtypes, tumor-infiltrating lymphocytes, and immune checkpoints expression. In sum, the findings of this study offer insightful strategies to guide the therapeutic direction, aiming at leveraging the impact of PDK family genes in cancer treatment.

## Introduction

Cancer, recognized as a pervasive global public health concern, has consistently held its position as the second leading cause of mortality worldwide, surpassed solely by cardiovascular disorders^1^. Despite substantial advancements in comprehending the pathogenesis, diagnosis, and treatment of cancer since the early twenty-first century, a comprehensive understanding of its biological characteristics and the establishment of an effective long-term management strategy remain elusive. Consequently, the annual incidence rate of cancer persists^2,3^. Although advancements in cancer therapy, including immunotherapy, targeted therapy, and radiation therapy, have been made, the 5-year overall survival (OS) rate for patients remains unsatisfactory^4,5^. Therefore, the development of novel cancer diagnostic and prognostic biomarker is crucial. To address these challenges, the ongoing development and refinement of public databases such as The Cancer Genome Atlas (TCGA) and Genotype-Tissue Expression (GTEx) have greatly facilitated the exploration of new immunotherapy targets. These databases provide invaluable resources for the discovery of innovative solutions to fight cancer.

The pyruvate dehydrogenase complex (PDC) stands as a crucial enzyme system in the body, facilitating the oxidative decarboxylation of pyruvate to generate acetyl coenzyme A. Positioned at a pivotal intersection between glycolysis and the tricarboxylic acid cycle, it plays a fundamental role in aerobic metabolism^6^. Phosphorylation of PDC is facilitated by four isoforms of pyruvate dehydrogenase kinase (PDK1–4), which exhibit approximately 70% homology in human^7^. These serine/threonine kinases are closely associated with PDC and bind to L2 domain in E2. Each PDK isoform possesses distinct specificities in terms of phosphorylating E1α and varies in rates of phosphorylation^8^. In mammals, PDK1–4 encoded by distinct genes and exhibit tissue- or cell-type-specific expression patterns, governing the precise regulation of PDC. These enzymes predominantly locate within the mitochondrial matrix and share up to 70% sequence homology, primarily differing in their N-terminal regions. PDK1 is primarily expressed in the heart, islet, and skeletal muscle, while PDK2 is ubiquitously distributed across various tissues, with exceptions in the spleen and lung. PDK3 is limited to the testes, kidneys, and brain, and PDK4 exhibits high expression levels in the heart, skeletal muscle, liver, kidneys, and islet^9^. Under normal physiological condition, pyruvate dehydrogenase phosphatase counteracts PDK, promoting the activation of PDC. This activation allows for the complete oxidation of pyruvate within the tricarboxylic acid cycle, thereby generating the essential ATP for the body’s energy demand. However, in specific pathological scenarios, the phosphorylation of PDC induces PDK activation. Consequently, PDC is inactivated, preventing the complete oxidation or conversion of pyruvate into fatty acid. Malignant cell transformation and alteration in metabolic pathways continually upregulate PDK. By inhibiting PDK and enhancing PDC activity, promising drug targets for therapeutic intervention can be identified.

In this study, our core focus revolved around dissecting the intricate structural aspects of PDK members. Through a comprehensive analysis, we explored the expression levels and prognostic implications associated with PDKs across various cancers, employing the rich resource of the TCGA and GTEx databases. Our research extended further to the interplay between PDKs expression and the dynamic landscape of the tumor microenvironment (TME). Moreover, our study encompassed an exploration of the correlation linking PDKs expression levels with distinct immune subtypes in multiple cancer types. Collectively, our study revealed the detailed PDK gene expression in pan-cancer. This view enabled us to unravel the intricacies of immune infiltration. Such insights hold promise, paving the way for PDKs to be considered as potent targets for therapeutic interventions in pan-cancer treatment strategies.

## Materials and methods

### PDK family data collection and processing

PDK gene family and associated clinical information from both tumor and corresponding normal samples were collected from The Cancer Genome Atlas (TCGA) and Genotype-Tissue Expression (GTEx) databases. UCSC Xena (https://xena.ucsc.edu/) was used for exploration of gene expression and clinical and phenotype data. The RNA sequencing data were Log2-transformed, and two sets of t-tests across various tumor types were conducted. Statistical significance was described as follows: *p < 0.05; **p < 0.01; ***p < 0.001. Analytical processes were performed using R software (Version 4.0.2) and enhanced the visual representation of the results were drawn using the R package “ggpubr”. Human Protein Atlas database (HPA, https://www.proteinatlas.org/) was utilized to explore PDKs mRNA distribution among different tissues. The violin images of the PDK in various pathological stages were required from the “Pathological Stage Plot” module of GEPIA.

### Prognostic value analysis of PDKs

The Kaplan–Meier (KM) plotter was employed to analyze the prognostic implication of PDKs across various cancer types. The “auto select best cutoff” model was chosen approach, involving the computation of all feasible cutoff values, and selection of the most optimal threshold. A single-variable Cox regression analysis was conducted to assess the significance of PDKs in predicting OS. R package “forestplot” was utilized to create forest plots for Cox regression analysis. A hazard ratio (HR) exceeding 1 and p-value less than 0.05, indicated a significant correlation between PDKs and an elevated risk of mortality.

### Mutation profile of the PDKs

The cBioPortal database provides resource for investigating and analyzing cancer genomics data including epigenetic, gene expression profile, and proteomics^10^. cBioPortal was used to dig into the landscape changes of the PDK family in pan-cancer.

### Immune infiltration analysis of PDKs

We used the CIBERSORT algorithm to analyze the composition of tumor-infiltrating immune cells, including 22 distinct immune cell types, across a dataset comprising 33 TCGA tumors. These immune cell types included regulatory T cells, follicular helper T cells, gamma delta T cells, naive CD4^+^ T cells, CD4^+^ memory-activated T cells, CD8^+^ T cells, follicular helper T cells, macrophages. Connection between immune infiltration and the degree of PDK family expression in distinct malignancies was assessed with the “CIBERSORT” algorithm and it was presented by heatmaps.

### Analysis of tumor mutation burden and microsatellite instability

Tumor mutation burden (TMB) serves as a crucial metric, representing the cumulative count of somatic coding mutation within a specific cancer type. This metric stands as a significant determinant of the efficacy of immunotherapy in various human cancer types^11^. Alteration data of all TCGA patients were downloaded from the UCSC XENA database. And TMB scores were calculated. Microsatellite instability (MSI) is a condition characterized by the presence of repetitive sequences of mono- and oligonucleotides (short tandem repeats). These sequences reflect DNA mismatch repair (MMR) deficiency. In parallel, MSI emerges as a marker for favorable response to immunotherapy. We obtained the MSI data from a recent study^12^. To explore the potential relationship between PDKs expression and both TMB and MSI, Spearman’s correlation coefficient was utilized.

### Correlation analysis of PDKs with immune checkpoint inhibitors (ICIs) biomarkers

Gene expression profiling within the TME holds the potential to evaluate active innate and adaptive immune response, offering insights into the identification of robust biomarkers for predicting the clinical efficacy of checkpoint inhibitor strategy^13^. To investigate this further, Spearman correlation analysis was utilized to explore the co-expression relationship between PDK genes and eight key immune checkpoint-related genes in pan-cancer, including PDCD1 (PD-1), CTLA4, CD274 (PDL1), PDCD1LG2 (PD-L2), HAVCR2, LAG3, SIGLEC15 and TIGIT. The analysis was carried out using R packages such as “limma” “reshape2”, and “RColorBrewer”. These findings were visually represented using heatmaps, and statistical significance was determined by a p-value less than 0.05.

### Correlation and enrichment analysis of PDKs

GeneMANIA is an online resource which can explore gene functionality, analyze gene cluster, and prioritize genes for functional assay^14^. In our study, GeneMANIA website was utilized to dissect and categorize the interaction between PDK genes and their related genes. WebGestalt is a free web platform exclusively designed for comprehensive enrichment analysis. WebGestalt has an array of enrichment analysis algorithms and offers abundant functional annotation databases. Within our study, WebGestalt database was used to execute GO and KEGG enrichment analysis for PDK gene set.

## Results

### Analysis of PDKs expression across pan-cancer and healthy tissues

**Figure 1:**
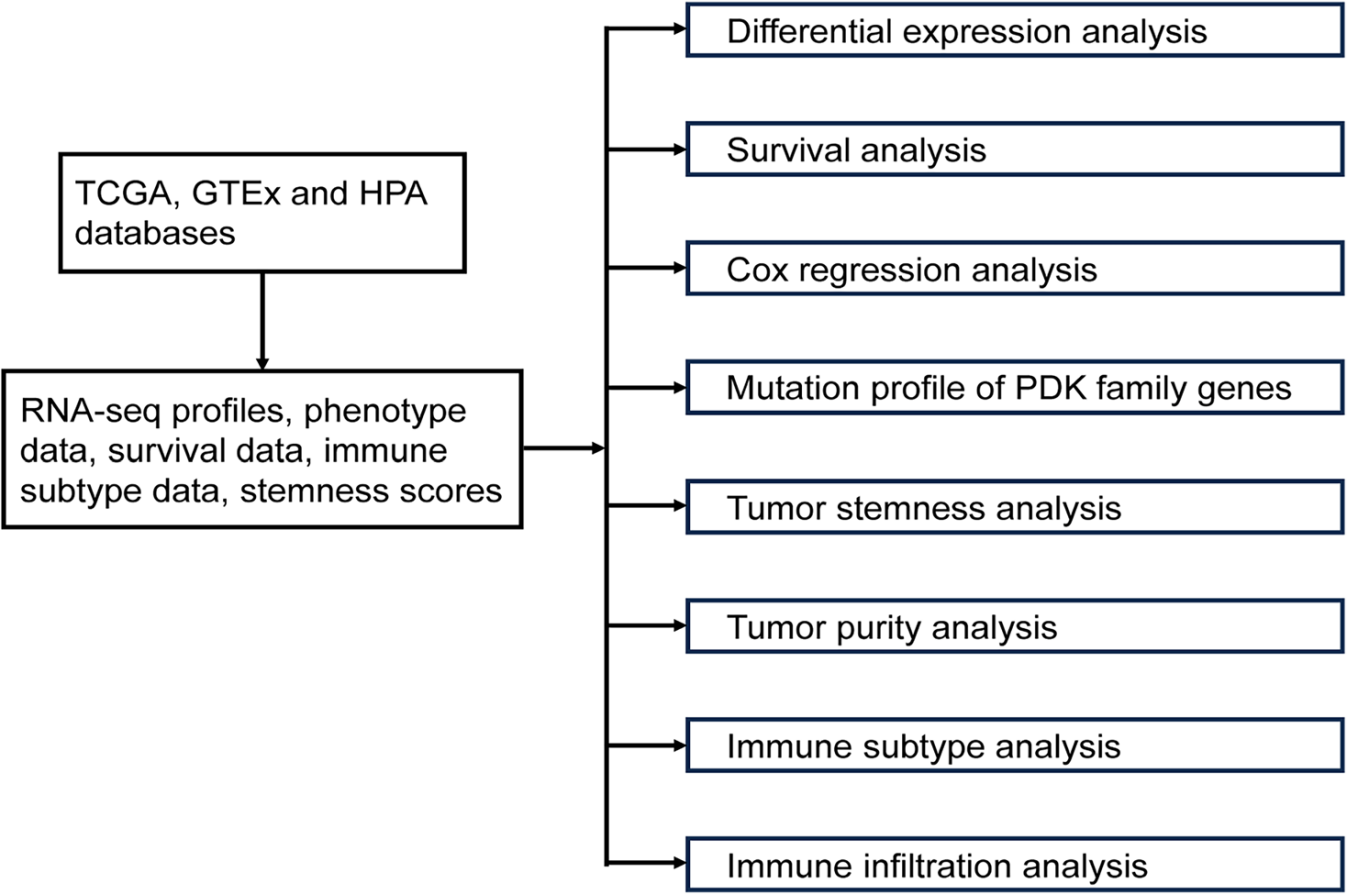
The flowchart of the whole analytic process of our study.

**Figure 2:**
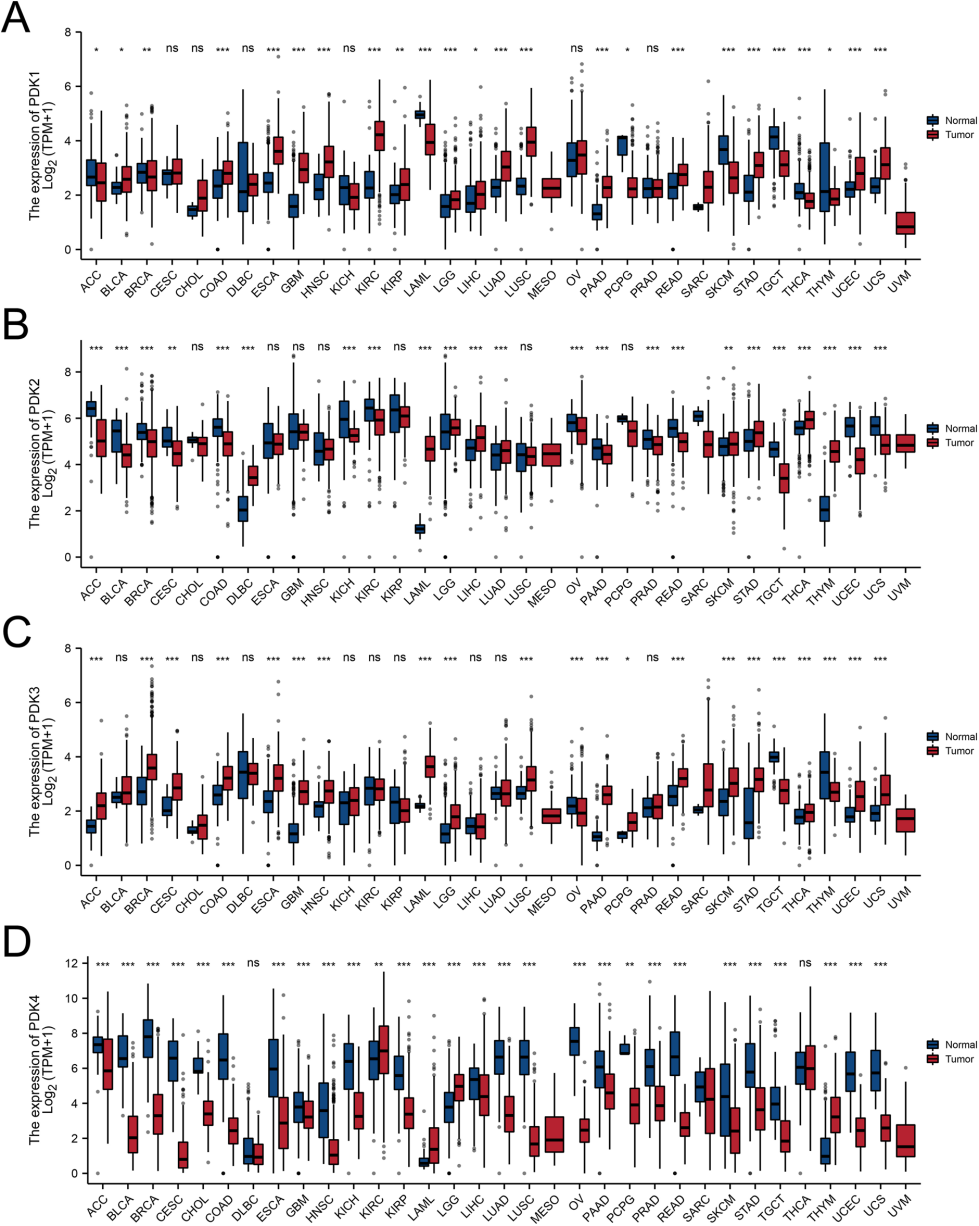
PDKs expression levels in different cancer types and normal samples. RNA-seq data were acquired from the TCGA and GTEx databases. (**A**) PDK1, (**B**) PDK2, (**C**) PDK3, (**D**) PDK4. *p < 0.05, **p < 0.01, and ***p < 0.001.

**Figure 3:**
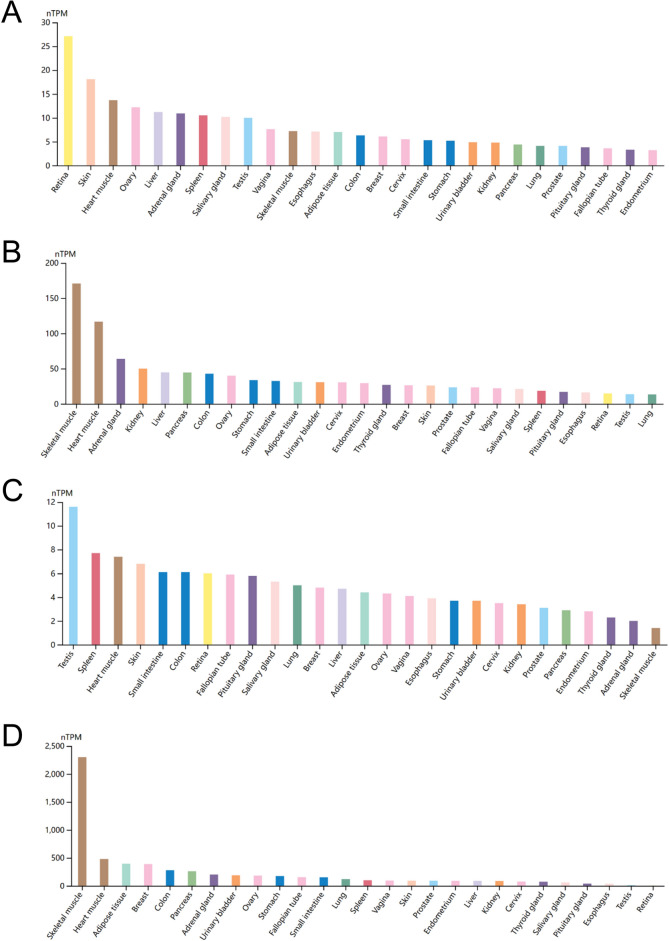
PDK family genes expression in normal human tissues from HPA database. (**A**) PDK1, (**B**) PDK2, (**C**) PDK3, (**D**) PDK4.

The analytical process of our study was displayed in Fig. 1. We conducted analysis of the expression pattern of the PDK family genes across a diverse range of cancer types using data from the TCGA and GTEx databases. By comparing the gene expression in tumors with their corresponding adjacent normal tissues, we uncovered notable discrepancies in the expression levels of PDK1, PDK2, PDK3, and PDK4. Figure 2 illustrated these patterns, for example, PDK1 exhibited consistent upregulation in BLCA (Bladder Urothelial Carcinoma), COAD (Colon adenocarcinoma), ESCA (Esophageal carcinoma), GBM (Glioblastoma multiforme), HNSC (Head and Neck squamous cell carcinoma), KIRC (Kidney renal clear cell carcinoma), KIRP (Kidney renal papillary cell carcinoma), LGG (Brain Lower Grade Glioma), LIHC (Liver hepatocellular carcinoma), LUAD (Lung adenocarcinoma), LUSC (Lung squamous cell carcinoma), PAAD (Pancreatic adenocarcinoma), READ (Rectum adenocarcinoma), STAD (Stomach adenocarcinoma), UCEC (Uterine Corpus Endometrial Carcinoma), and UCS (Uterine Carcinosarcoma). Conversely, PDK1 expression demonstrated a concurrent reduction in ACC (Adrenocortical carcinoma), BRCA (Breast invasive carcinoma), LAML (Acute Myeloid Leukemia), PCPG (Pheochromocytoma and Paraganglioma), SKCM (Skin Cutaneous Melanoma), TGCT (Testicular Germ Cell Tumors), THCA (Thyroid carcinoma), and THYM (Thymoma). Notably, elevated PDK2 expression was observed exclusively in DLBC (Lymphoid Neoplasm Diffuse Large B-cell Lymphoma), LAML (Acute Myeloid Leukemia), LGG, LIHC, LUAD, SKCM, STAD, THCA, and THYM. PDK2 expression was significantly downregulated in a multitude of cancers including ACC, BLCA, BRCA, CESC (Cervical squamous cell carcinoma and endocervical adenocarcinoma), COAD, KICH (Kidney Chromophobe), KIRC, OV (Ovarian serous cystadenocarcinoma), PAAD, PRAD (Prostate adenocarcinoma), READ, TGCT, UCEC, and UCS. In contrast, PDK3 exhibited widespread upregulation in most cancer types, except for OV, TGCT, and THYM. Intriguingly, PDK4 demonstrated a universal decrease across malignancies, except for KIRC, LAML, LGG, and THYM, where it exhibited an increase. Examine the HPA database, we revealed diverse expression profile of PDK genes in healthy tissues. As shown in the histograms in Fig. 3, PDK1 displayed heightened expression in retina and skin, while PDK2 and PDK4 were prominently expressed in skeletal muscle and heart muscle. PDK3 expression level was elevated in testis and spleen.

Furthermore, normal breast epithelial cell line (MCF-10A) and three distinct breast cancer (BC) cell lines characterized by different receptor expression were chosen: HER2^+^ BC cell line BT-474, ER^+^ BC cell line MCF-7, and triple-negative BC cell line BT-549. RT-qPCR experiments were conducted to assess the expression levels of PDKs. In Supplementary Fig. 1, it is evident that PDK1 exhibited lower expression in MCF-7 and BT-474. The mRNA expression level of PDK2 was higher in three breast cell lines than that in MCF-10A. On the contrary, PDK3 expression was lower in three breast cell lines than that in MCF-10A. PDK4 expression was downregulated in BT-474 and BT-549.

Expanding our investigation to the TCGA database, we delved into correlation among PDKs in various cancer types. PDK1 and PDK4 emerged as highly expressed genes in KIRC (Supplementary Fig. 2A). A more comprehensive examination across pan-cancer unveiled distinct expression pattern. PDK4 showed heightened expression, while PDK1 demonstrated lower expression level. Other members of the PDK family exhibited moderate expression across pan-cancer dataset (Supplementary Fig. 2B). When considering all 33 cancer types, PDK1 exhibited a significant positive correlation with PDK3. PDK2 displayed a positive association with PDK4 (Supplementary Fig. 2C).

To enhance the depth of our investigation, we applied the “Pathological Stage Plot” module within GEPIA2. This enabled us to explore the interplay between the expression of the PDKs and the pathological stages across a diverse array of cancers. PDK1 expression exhibited noteworthy variation tied to different stages in LIHC (Supplementary Fig. 3A). Meanwhile, a negative correlation between PDK1 expression and advanced tumor stages in OV and THCA was observed. Similar negative correlation with advanced tumor stages was found in PDK2, particularly evident in KIRC (Supplementary Fig. 3B). PDK3 exhibited staging-specific expression in LIHC, PAAD, SKCM, TGCT and THCA (Supplementary Fig. 3C). And PDK4 displayed staging-specific expression in BLCA, BRCA, CESC, ESCA, KIRC, LUSC, PAAD, SKCM and STAD (Supplementary Fig. 3D).

### Prognostic value of PDKs in pan-cancer

**Figure 4:**
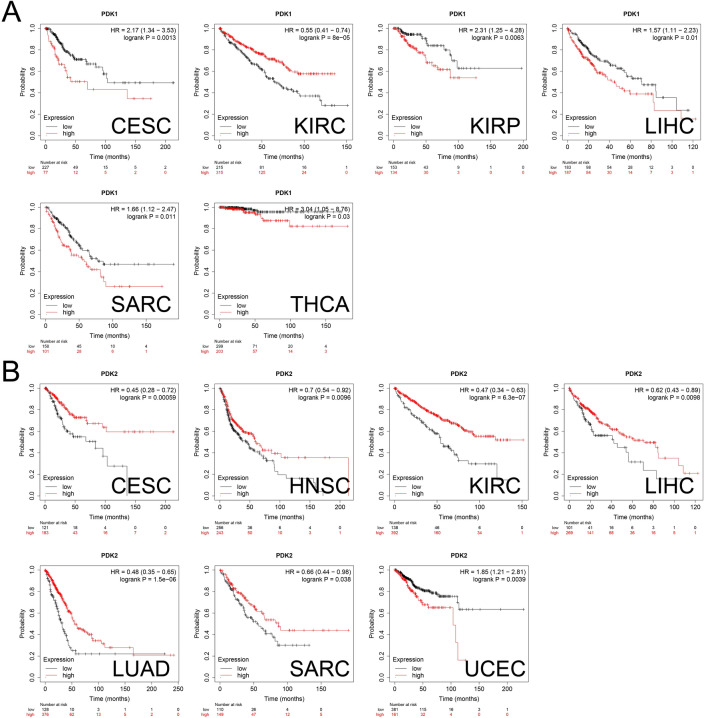
Correlation between OS and (**A**) PDK1 and (**B**) PDK2 expression levels in different cancers. The curves generated through the use of KM plotter database display the prognostic value of PDK family genes. The red lines represent high PDKs expression, while the black lines represent low PDKs expression.

**Figure 5:**
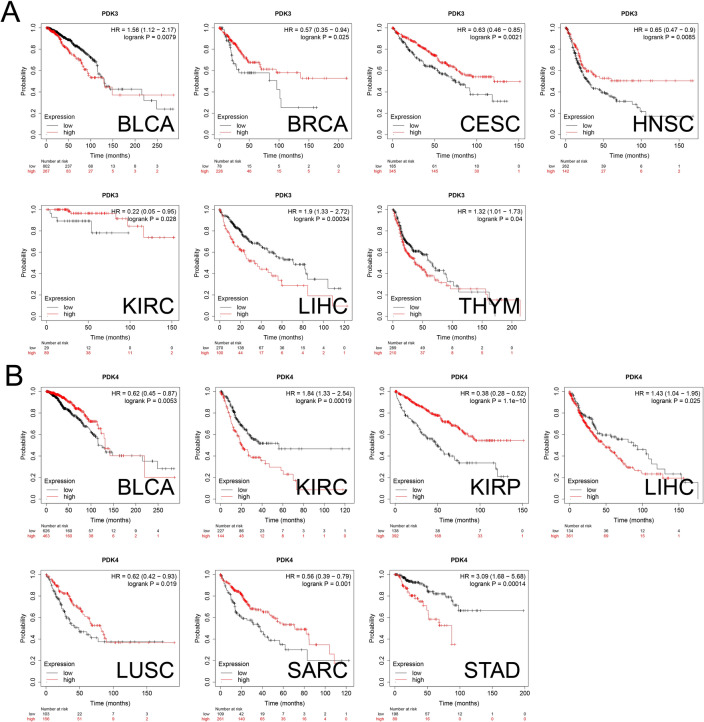
Correlation between OS and (**A**) PDK3 and (**B**) PDK4 expression levels in multiple malignances.

Seeking to investigate the intricate connection between the expression levels of PDKs and their potential impact on prognosis of cancer patients, our study undertook comprehensive survival analysis using Kaplan–Meier survival analysis. High levels of PDK1 were associated with worse OS in CESC, KIRP, LIHC, SARC (Sarcoma) and THCA, and better clinical outcome was found in KIRC patients (Fig. 4A). Patients with high PDK2 expression in CESC, HNSC, KIRC, LIHC, LUAD and SARC experienced improved clinical endpoint, while worse prognosis was found in UCEC patients (Fig. 4B). High expression of PDK3 was linked to worse OS in BLCA, LIHC and THYM, while it acted as a beneficial factor in BRCA, CESC, HNSC and KIRC (Fig. 5A). High expression of PDK4 was linked to better endpoint in BLCA, KIRP, LUSC and SARC, while it posed a high-risk factor for patients with KIRC, LIHC and STAD (Fig. 5B).

**Figure 6:**
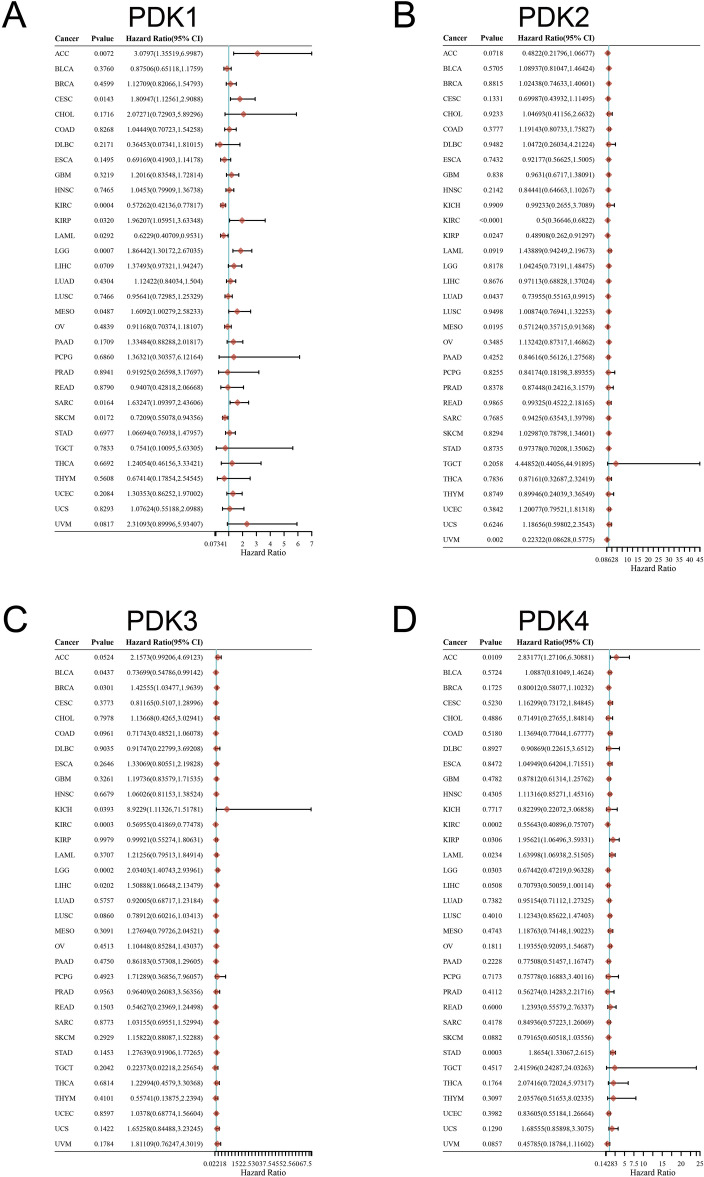
The univariate cox regression analysis between PDKs expression and patient’s overall survival of different cancers (**A**) PDK1, (**B**) PDK2, (**C**) PDK3, (**D**) PDK4. The hazard ratios and 95% confidence intervals in the forests demonstrating the survival advantage and disadvantage about high expression of PDKs.

Next, we explored the prognosis value of PDKs using COX analysis. As shown in Fig. 6A, PDK1 emerged as a protective factor in cases of KIRC, LAML, and SKCM. However, PDK1 took on a high-risk role in ACC, CESC, KIRP, LGG, MESO (Mesothelioma), SARC. PDK2 had protective role in KIRC, KIRP, LUAD, MESO and UVM (Uveal Melanoma) (Fig. 6B). PDK3 was a low-risk factor in BLCA and KIRC. However, PDK3 exhibited detrimental effect in BRCA, KICH, LGG, and LIHC (Fig. 6C). PDK4 acting as a high-risk gene in ACC, KIRP, and LAML, while acting protectively in KIRC, LGG, STAD (Fig. 6D). These findings indicated the intricate roles that PDKs played in different cancers, illuminating their diverse influence on prognosis.

### The mutation profile of PDKs

**Figure 7:**
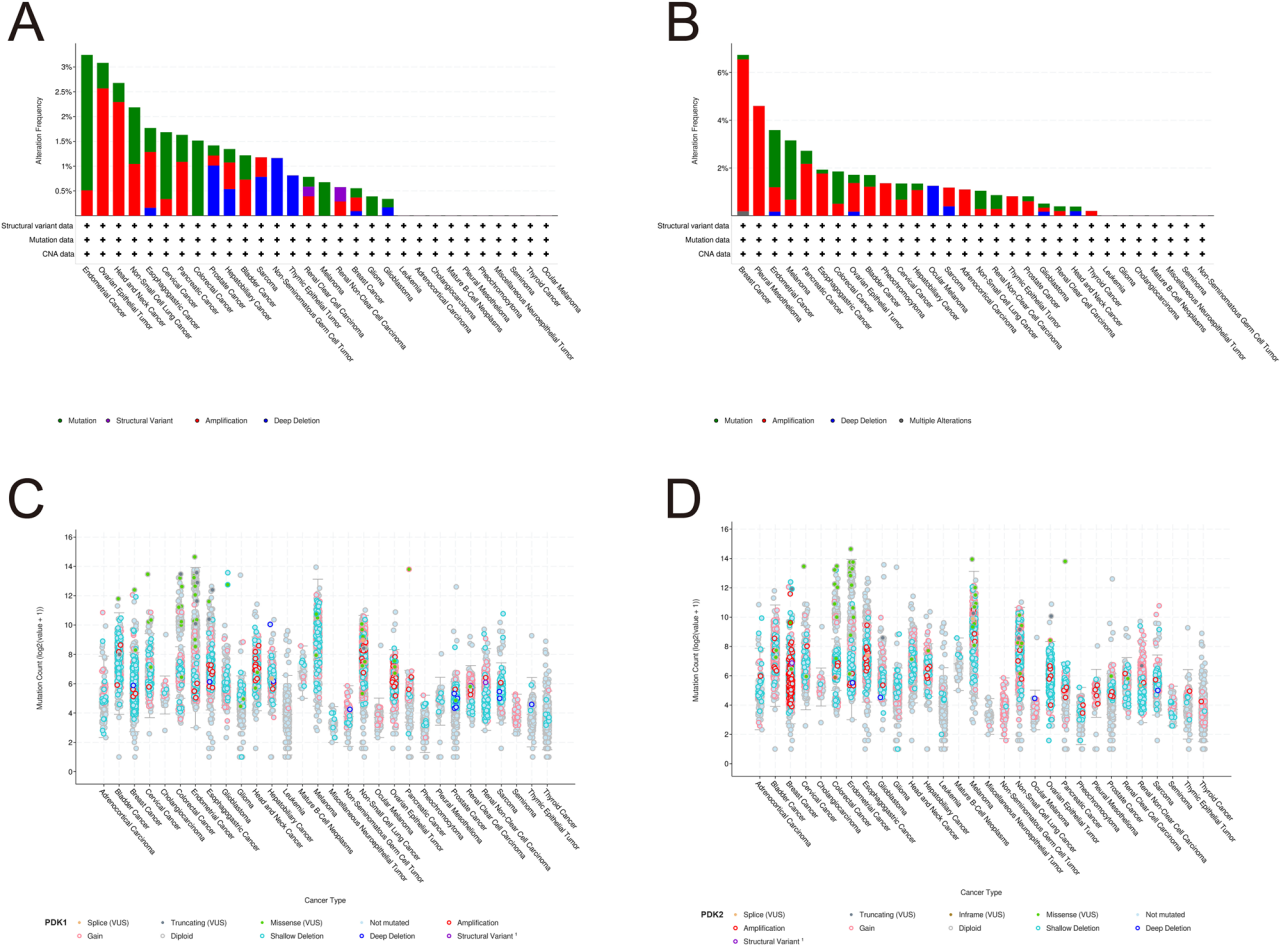
PDK1 and PDK2 mutation landscape. (**A,B**) PDK1 and PDK2 mutation frequency in various TCGA cancer types based on the cBioPortal database. (**C,D**) The general mutation count of PDK1 and PDK2 in multiple types of cancers according to the cBioPortal database.

We utilized the cBioPortal platform to conduct an in-depth analysis of the mutation pattern about the PDK family genes. To summarize our findings, we observed that the occurrence of mutation in the four PDK genes remained below 5% in majority of cancers cases. As displayed in Fig. 7A, the top five cancer types with alteration in PDK1 were UCEC, SARC, mature B cell neoplasms, melanoma, and esophagogastric cancer. Similarly, the top five cancers showing PDK2 alteration were esophagogastric cancer, head and neck cancer, melanoma, mature B cell neoplasms, and UCEC. The principal mutation type of PDK2 was amplification (Fig. 7B). While shallow deletion of PDK1 was widespread across various cancers, gain in the PDK2 was prevalent (Fig. 7C,D).

**Figure 8:**
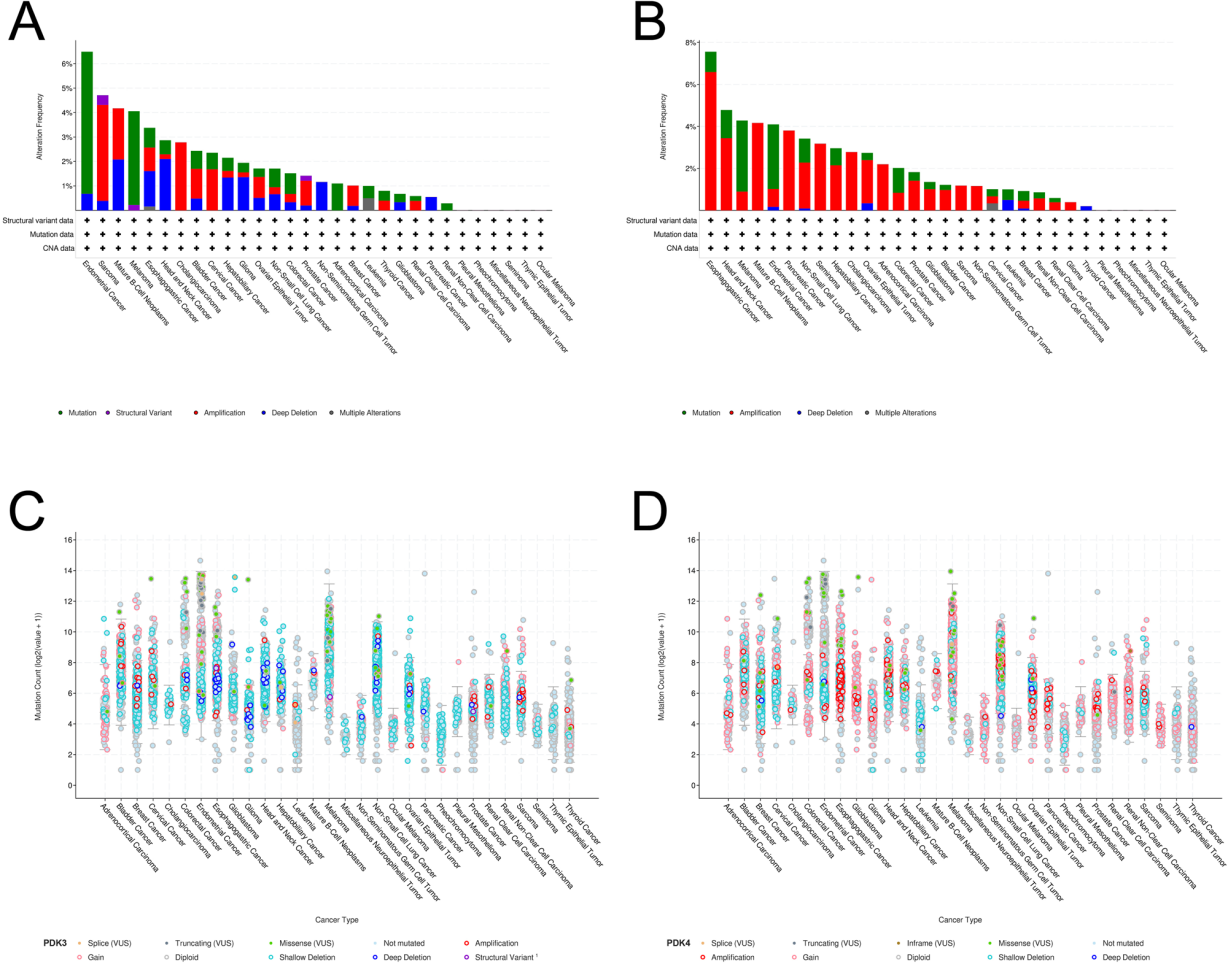
PDK3 and PDK4 mutation landscape. (**A,B**) PDK3 and PDK4 mutation frequency in various TCGA cancer types based on the cBioPortal database. (**C,D**) The general mutation count of PDK3 and PDK4 in multiple types of cancers according to the cBioPortal database.

Moving to the alteration of PDK3, we identified the leading cancer types with PDK3 mutation as UCEC, ovarian epithelial tumor, head and neck cancer, non-small cell lung cancer, and esophagogastric cancer (Fig. 8A). As for PDK4 mutation, the top five cancer categories included breast cancer, pleural mesothelioma, UCEC, melanoma, and PAAD (Fig. 8B). The primary alteration of PDK3 was shallow deletion, and gain of PDK4 was prevalent across various cancers (Fig. 8C,D).

### Relationship between PDKs expression and tumor microenvironment

**Figure 9:**
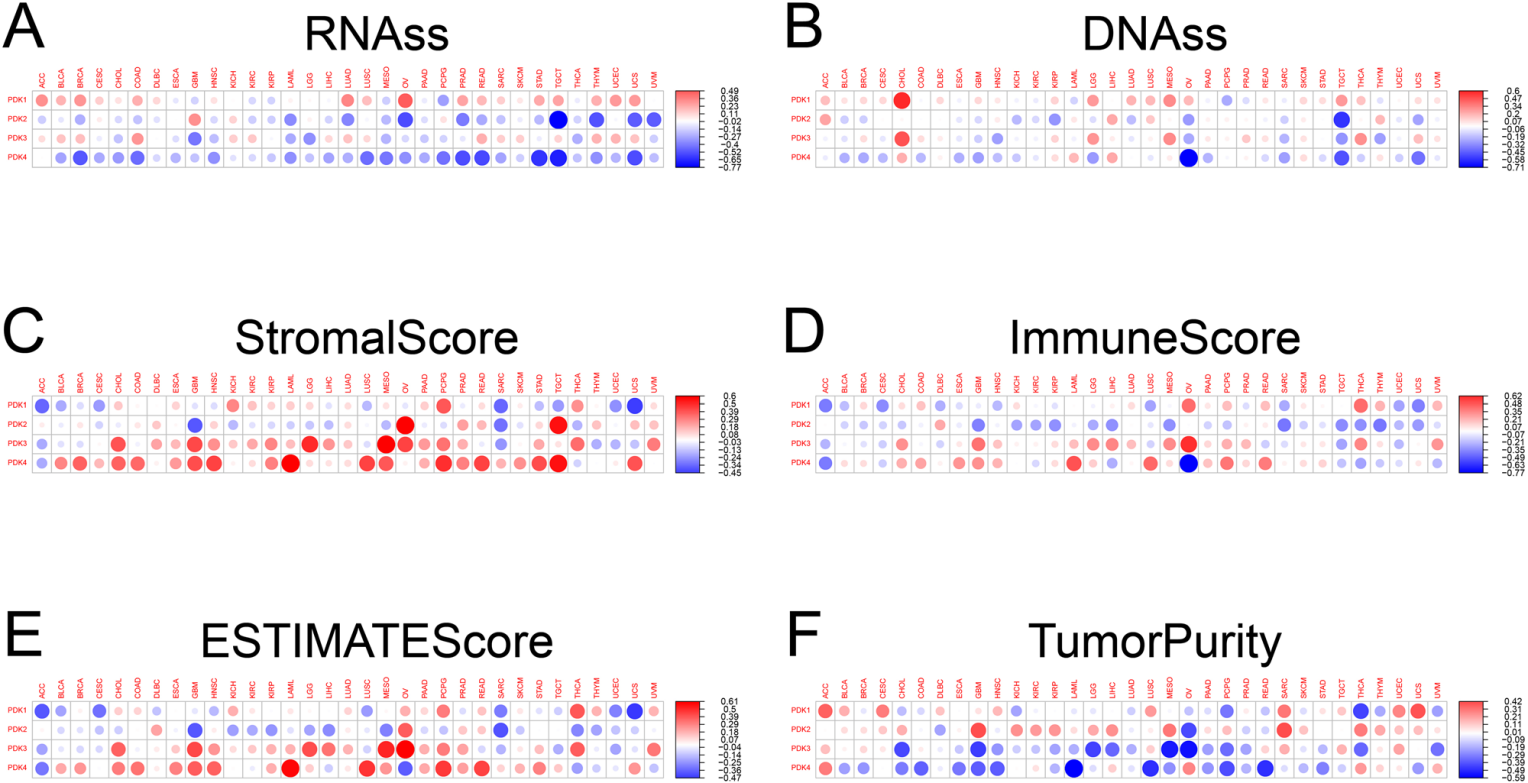
Relationship between tumor microenvironment and PDKs expression in pan-cancer. (**A,B**) PDKs expression is correlated with RNAss and DNAss in multiple malignances. The Spearman correlation coefficient is employed for statistical analysis. The magnitude of the dot reflects the absolute value of the correlation coefficients, with larger dots indicating higher correlation strength. Red dots indicate a positive correlation and blue dots indicate a negative correlation. (**C–E**) PDKs expression is correlated with estimate score, immune score, and stromal score in different cancers. (**F**) PDKs expression is related to tumor purity.

Cancer stem cells is key player in multiple tumor-related processes. These processes include tumor proliferation, migration, metastasis, tumor angiogenesis, epithelial-to-mesenchymal transition, and resistance to therapeutic intervention^15^. To comprehensively investigate the intricate interplay between the expression of PDKs and the extent of cellular stemness, we conducted an in-depth analysis. Figure 9A,B revealed a distinctive pattern of association between PDKs expression and the levels of DNA stemness score (DNAss) and RNA stemness score (RNAss) across diverse cancer types. For example, in various malignancies, the expression of PDK4 showed a consistent inverse relationship with RNAss. In addition, all PDK genes displayed a negative correlation with RNAss in MESO. Moreover, PDK1 expression manifested a positive association with DNAss in OV and TGCT, while PDK2, PDK3, and PDK4 exhibited significant negative correlation with DNAss in OV and TGCT.

Within the dynamic of the TME, two important non-tumor components, immune cells and stromal cells, emerge as significant players with prognostic biomarkers of cancer patients^11^. The quantified scores derived from ImmuneScore and StromalScore offer insights into the proportion of immune and stromal components within the TME. The amalgamated ESTIMATEScore provides a comprehensive overview of both components in tandem. The relationship between PDKs expression and these scores was displayed in Fig. 9C–E. The expression of PDK2 showed a positive correlation with StromalScore in TGCT, THYM, UCS, and UVM, while concurrently exhibiting a negative correlation with ImmuneScore in above tumor types. PDK3 showed consistent positive association with StromalScore and ESTIMATEScore in various tumors, including CHOL (Cholangio carcinoma), ESCA, GBM, HNSC, KIRC, KIRP, LAML, LGG, LIHC, LUAD, MESO, OV, PAAD, PCPG, PRAD, STAD, THCA, and UVM. PDK4 was significantly positively linked to StromalScore and ESTIMATEScore in multiple cancers, including BLCA, BRCA, CHOL, COAD, ESCA, GBM, HNSC, KIRP, LAML, LUSC, MESO, PAAD, PCPG, PRAD, READ, SKCM, STAD, and UCS. Remarkably, both PDK3 and PDK4 expression profile was inversely associated with tumor purity across a majority of cancers (Fig. 9F). These results demonstrated the distinctive roles of PDK family members in orchestrating the intricate immune microenvironment within divergent cancers.

### Correlation between immune infiltration and PDKs expression

**Figure 10:**
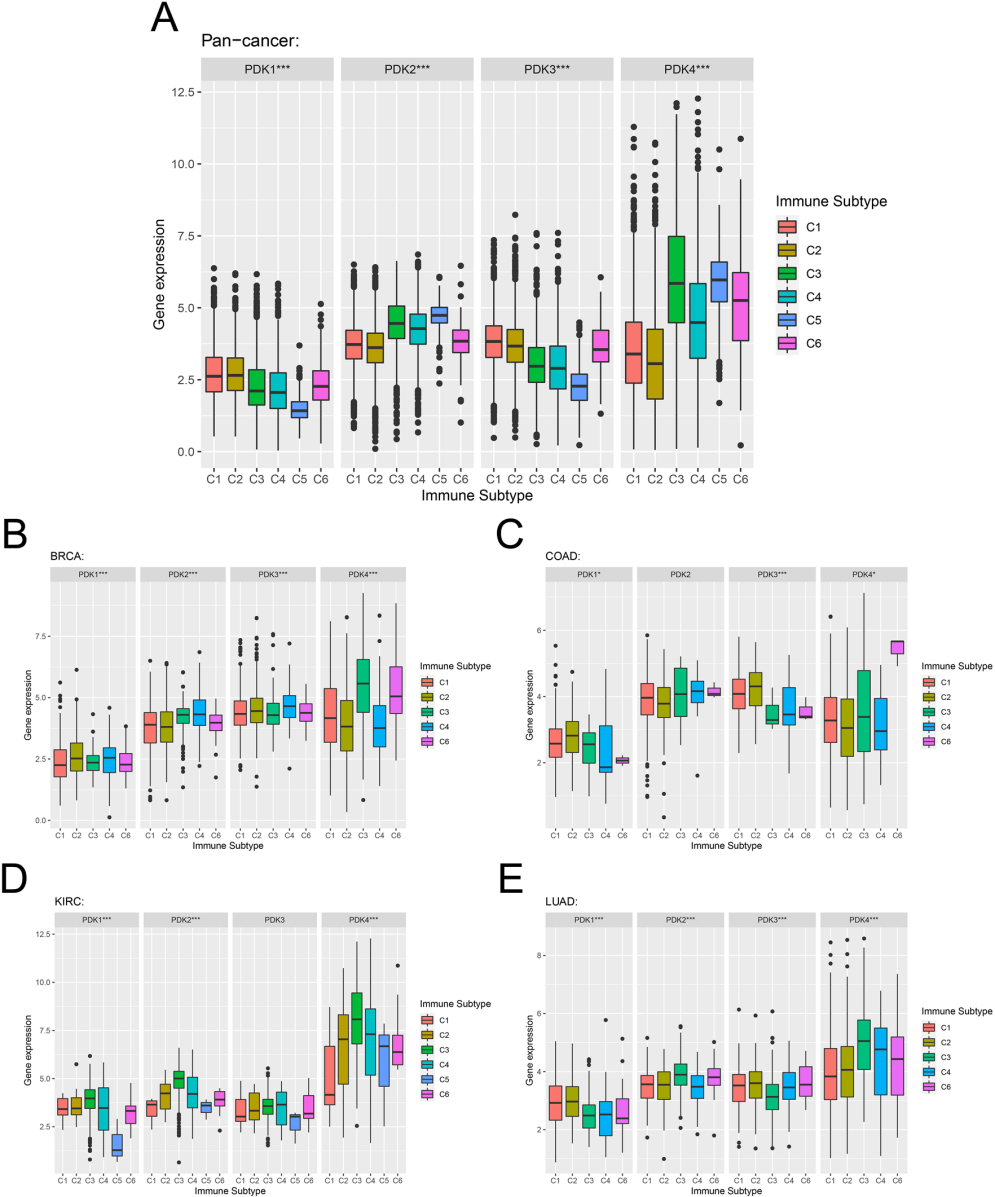
PDKs expression levels of different immune subtypes in (**A**) pan-cancer, (**B**) BRCA, (**C**) COAD, (**D**) KIRC, (**E**) LUAD. C1: wound healing, C2: IFN-gamma dominant, C3: inflammatory, C4: lymphocyte depleted, C5: immunological quiet, and C6: TGF-beta dominant. X axis indicates immune subtype, Y axis indicates gene expression. *p < 0.05, ***p < 0.001.

The relationship between PDKs and the immune subtypes was analyzed to elucidate their potential significance in various cancers. Figure 10A demonstrated the significant connection between the expression levels of PDK family members and different immune subtypes: C1 (wound healing), C2 (IFN-gamma dominant), C3 (inflammatory), C4 (lymphocyte depleted), C5 (immunologically quiet) and C6 (TGF-b dominant) in pan-cancer. Then, we focused on BRCA, COAD, LUAD, and KIRC, revealing intriguing pattern. PDK1 was found to be highly expressed in C2 and C4, while PDK2 and PDK3 exhibited upregulation in C4 and PDK4 demonstrated elevated expression in C3 (Fig. 10B). Furthermore, in COAD, PDK1, PDK3, and PDK4 expression was significantly linked to immune subtype variation (Fig. 10C). Similarly, we observed significant difference in KIRC, where PDK1, PDK2, and PDK4 were overexpressed in C3 (Fig. 10D). In LUAD, PDK1 and PDK3 were elevated in C2, whereas PDK2 and PDK4 expression was more pronounced in C3 (Fig. 10E). These findings reinforced the distinct roles of PDK members within diverse tumors.

**Figure 11:**
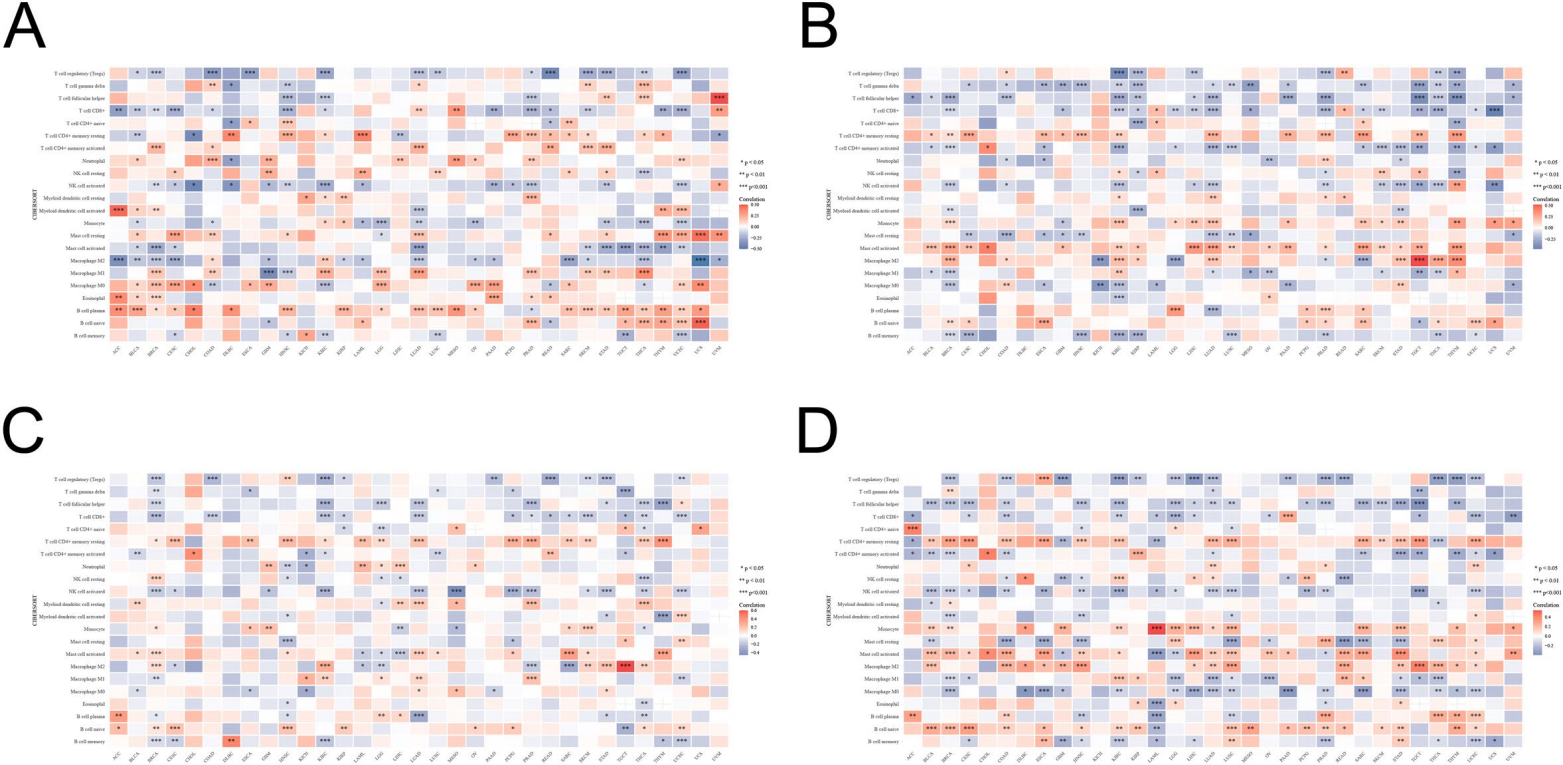
The relationship between infiltration levels of different immune cells and expression of (**A**) PDK1, (**B**) PDK2, (**C**) PDK3, (**D**) PDK4 based on cibersort algorithm. *p < 0.05, **p < 0.01, ***p < 0.001.

Recognizing the important role of tumor-infiltrating immune cells in the TME, which influences cancer initiation, progression, and immunotherapy response^16,17^, we explored the relationship between immune cell infiltration and the expression of PDKs across different cancer types. Using the RNA-sequencing profile from the TCGA dataset and employing the “CIBERSORT” algorithm, we assessed the interplay between PDKs expression and the levels of infiltration of 22 distinct immune cell types. As displayed in Fig. 11A, PDK1 expression exhibited a positive correlation with plasma cell, while it was negatively associated with activated mast cell. Notably, PDK1 was significantly negatively correlated with M2 macrophage and positively associated with M1 macrophage in BRCA, LUAD, SKCM and THCA. PDK2 expression was negatively correlated with gamma delta T cell and follicular helper T cell across a variety of tumors (Fig. 11B). In addition, PDK2 exhibited a significant positive association with resting CD4^+^ T cell and a negative correlation with activated CD4^+^ T cell in BLCA, BRCA, ESCA, KIRC, LUAD, SARC, TGCT and THYM. Furthermore, PDK2 was also positively associated with M2 macrophage and negatively correlated with M1 macrophage in BRCA, LUAD and SARC. For PDK3 expression, the results revealed a negative correlation with CD8^+^ T cell and activated NK cell (Fig. 11C). Meanwhile, it exhibited a significant positive association with M1 macrophage and negative correlation with M2 macrophage in LGG and PRAD. An opposite relationship between PDK3 and macrophage was found in BRCA. Examining PDK4 expression, there was a negative correlation with infiltrating levels of follicular helper T cell, activated NK cell and M0 macrophage (Fig. 11D). In addition, PDK4 was positively correlated with M2 macrophage and negatively associated with M1 macrophage in LUAD, STAD, TGCT and THCA.

Furthermore, our investigation extended to coexpression analysis, revealing the interaction between PDKs expression and main immune checkpoints across pan-cancer (Supplementary Fig. 4). PDK1 exhibited positive correlation with representative immune checkpoints in THCA and LUAD, while PDK3 displayed varying association in different cancer types. PDK3 expression was negatively associated with TGCT and significantly positively correlated with PRAD, LUAD, and HNSC. This result indicated potential relevance of the PDK family genes in immunotherapy.

Considering the clinical importance of TMB and MSI in assessing the efficacy of immunotherapy^18,19^, we explored the correlation between the PDKs and TMB/MSI. Our analysis demonstrated distinct association between TMB and the expression of PDK1, PDK2, PDK3, and PDK4 in various cancer types (Supplementary Fig. 5A–D). Additionally, the relationship between PDKs expression and MSI was examined and displayed in Supplementary Fig. 6.

### Correlation and enrichment analysis of PDKs

**Figure 12:**
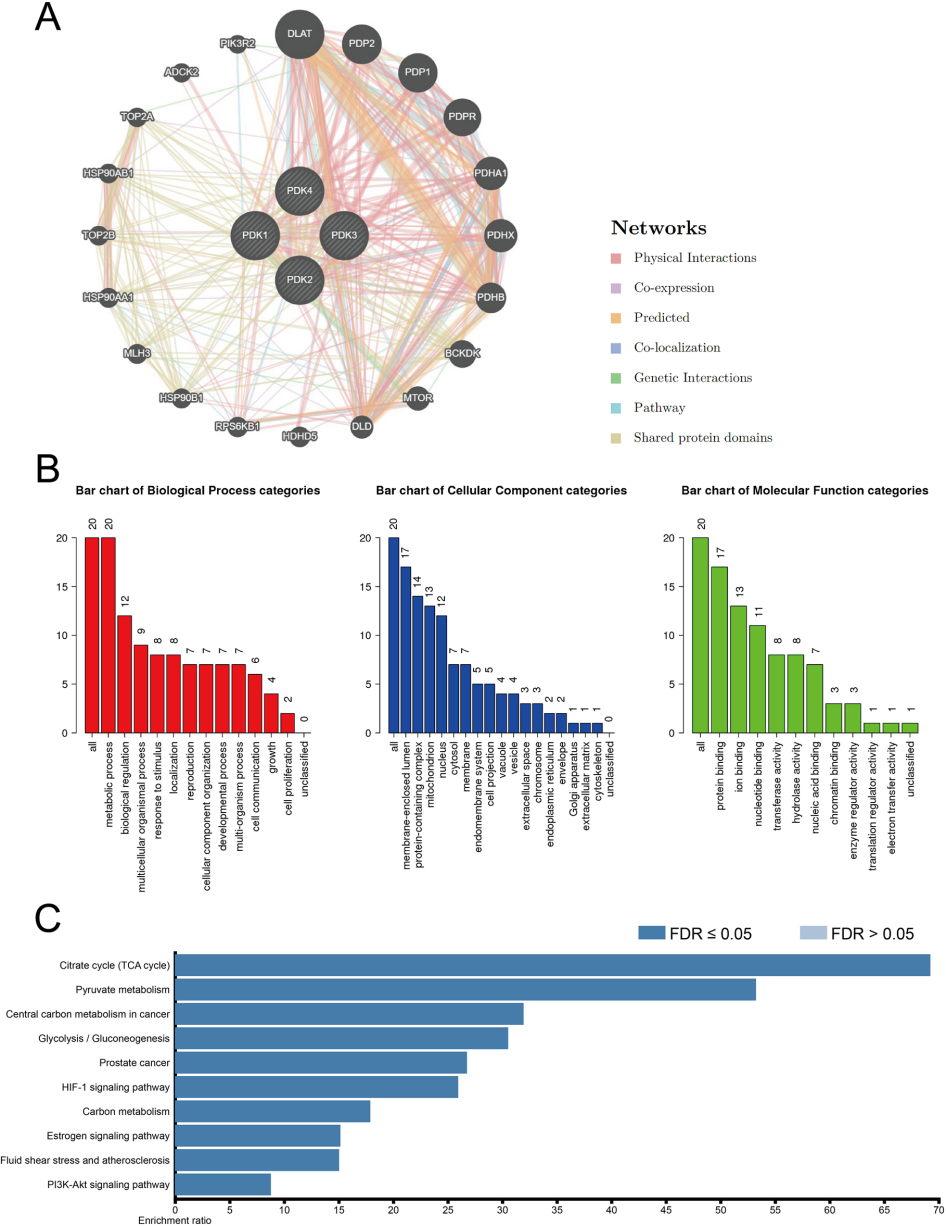
Enrichment analysis of the associated genes of PDKs. (**A**) Protein–protein interaction network of the PDKs conducted by using GeneMANIA. (**B**) GO analysis of relevant biological processes, cellular components, and molecular functions of the genes related to PDKs. (**C**) KEGG analysis revealing the related signal pathways.

Subsequently, we utilized the GeneMANIA tool to identify potential interaction partners of PDKs. This outcome unveiled a compelling correlation between PDKs expression and an array of noteworthy candidates, prominently including DLAT, PDP2, and PDP1, among others (Fig. 12A). To further amplify our understanding, the WebGestalt database was used to conduct GO and KEGG enrichment analysis on the cohort of 20 genes linked to PDKs. The GO analysis illuminated the involvement of these genes in fundamental biological processes, including metabolic processes and biological regulation. The KEGG analysis result showed the citrate cycle, pyruvate metabolism, and central carbon metabolism in cancer as the top three enriched pathways, suggesting important roles for these genes in the intricate machinery of cell metabolic process (Fig. 12B,C).

## Discussion

Tumor cells manifest distinct hallmarks characterized by their resistance to apoptosis, heightened signaling pathways, accelerated proliferation, invasive potential, promotion of angiogenesis, and metabolic alterations^20^. Altered glucose metabolism stands out as a prevalent trait in tumor cells. Typically, glucose, a pivotal energy substrate, undergoes oxidation in mitochondria through oxidative phosphorylation (OXPHOS) to yield ATP. Under hypoxic conditions, OXPHOS activity diminishes, prompting the initiation of the glycolytic pathway. Remarkably, tumor cells often exhibit a preference for glycolysis over efficient mitochondrial respiration, a phenomenon recognized as the “Warburg effect,” even in normoxic environment where oxygen is abundant^21^. This metabolic alteration leads to an increased demand for glucose to compensate for the inefficient ATP production from glycolysis^22^. Such adaptation provides cancer cells with a survival advantage in hypoxic conditions, shielding them from oxidative stress and apoptosis, consequently fueling uncontrolled growth and metabolism. At the crux of this metabolic shift lie the PDKs and PDC. The involvement of PDK family genes has been implicated in numerous cancers^23–25^. While the association between PDKs expression and pathological progression is well-established in certain tumors, a comprehensive analysis across different cancers has been lacking. Our study pioneers in exploring the expression and prognostic significance of PDK family genes in human tumors, thereby unveiling the mechanisms that underlie tumor progression.

Significant focus has been devoted to PDK1 in the realm of cancer research. PDK1 is frequently associated with HIF1α expression, which is modulated by either hypoxic or normoxic conditions, wherein HIF1α serves as a direct regulator of PDK1^26,27^. Furthermore, hypoxia can modulate PDK1 via the accumulation of Akt2 in mitochondria, leading to Thr346 phosphorylation of PDK1, a novel posttranslational modification that independently controls PDK activity^28^. HIF1α deficiency markedly decreases cell growth under hypoxia, a deficit that can be almost fully reversed by PDK1 overexpression. Earlier studies on PDKs in cancer indicated that the elevation of HIF1α stabilized PDK1 activity in head and neck squamous cancer cells (HNSCC). Notably, PDK1 knockdown not only reverses the Warburg effect but also reduces invasiveness and tumor growth^27^. Similar trends have been documented in other cancer types, including clear cell renal carcinoma, breast cancer, among others^29,30^. For instance, robust expression of PDK1 in renal cell carcinoma tissues correlates with enhanced cell proliferation, migration, invasion, and EMT, all regulated by the PI3K-PDK1-Akt pathway^31^. Similar pattern emerges in hepatocellular carcinoma (HCC), where high PDK1 expression correlates with unfavorable postoperative outcome^32^. Our study represents the first exploration of PDK1 expression and prognostic value across pan-cancer. Using TCGA and GTEx datasets, our findings showed PDK1 as a potential biomarker for predicting tumor prognosis.

The ubiquitous expression of PDK2 across various tissues renders it a focus of multiple cancer studies^33^. It is the sole PDK enzyme so far confirmed as a p53 target, possibly a mechanism by which WT p53 modulates metabolism^34^. Heightened PDK2 expression is associated with a worse prognosis, while inhibiting this enzyme has shown promise in boosting cisplatin sensitivity. This effect is achieved by promoting electron transport chain activation and intensifying the generation of mitochondrial reactive oxygen. Mouse xenograft models have demonstrated that combining cisplatin treatment with PDK2 inhibition effectively inhibits tumor growth^35^. High PDK2 expression was associated with a favorable clinical outcome in specific cancers like CESC, HNSC, KIRC, LIHC, LUAD, and SARC, suggesting its protective role.

While less explored, the induction of PDK3 by HIF1α has been documented, and its forced expression increases glycolysis and drug resistance in multiple cancer cell lines^36^. The connection of PDK3 to the histone lysine demethylase JMJD2A–E2F1 complex and its role in KDM4A-induced tumorigenesis have been established^24^. PDK4, primarily expressed in muscle and liver tissues but also found in other epithelial cells, is regulated by multiple transcription factors including PPAR-α, HIF1α, PPAR-gamma, and farnesoid X receptor^37^. Past studies suggest that the inhibition of miR-5683 promotes glycolysis by upregulating PDK4 expression, thereby driving gastric cancer progression^38^. Inhibiting PDK4 has emerged as a pivotal theme in combating drug resistance. Inhibiting PDK4 in tamoxifen-resistant breast cancer cells sensitizes them to tamoxifen/fulvestrant treatment, presenting a promising therapeutic strategy^39^. Activation of PPAR-gamma by pioglitazone induces PDK4 expression, leading to a reduction in cancer cell outgrowth in a PDK4-dependent manner. The ROS production prompted by pioglitazone has been linked to PDK4 expression^40^. Additionally, the impact of PDK4 goes beyond growth control, encompassing enhanced migration, invasiveness, activation of the epithelial–mesenchymal transition, and increased metastasis rates through its regulation of mitochondrial energy production^41^. We add to this body of knowledge by revealing the negative impact of PDK4 on clinical outcome in STAD patients.

The hallmarks of cancer, including sustained proliferative signaling and evasion of immune response, rely to varying degrees on the TME^42^. This TME dependence presents opportunities for therapeutic intervention via TME elements or signaling pathways. Our study highlights the close link between PDKs expression and immune subtypes and ESTIMATEScore of diverse tumors, suggesting the significant role of PDKs in TME. A critical player in maintaining self-tolerance is the regulatory T cell (Tregs), characterized by the expression of Foxp3. These cells play a central role in suppressing immune response directed at self or foreign antigens^43,44^. In tumors, Tregs infiltrating the tumor microenvironment can yield unfavorable clinical outcome, driving tumor progression by hampering antitumor immune response and promoting angiogenesis^45^. Consequently, they pose a significant impediment to the effective application of cancer immunotherapy. In our study, PDKs positively correlate with immune checkpoint genes expression and negatively correlated with Tregs infiltration in several tumors, including LUAD and THCA, suggesting their potential as favorable prognostic markers for immunotherapy.

The prevalence of tumor-associated macrophages (TAMs) within the TME makes them a pivotal player in tumor immune response. TAMs can polarize into M1 or M2 subtypes, with opposing effect on tumor progression^46^. The M1-like macrophages exhibit an antitumor profile by releasing proinflammatory cytokines, such as TNF and interleukin-2, along with generating reactive nitrogen and oxygen intermediates^47^. Conversely, the M2-like macrophages, activated by cytokines such as IL-4, IL-10, and IL-13 from type 2T helper cell (Th2), actively foster tumor growth. Our findings reveal connection between the expression of PDK1 and PDK3 with the M1 subtype of macrophages, showing a positive correlation, while a converse association emerges with the M2 macrophages across various tumor types. In parallel, intriguingly, an opposing pattern was found between PDK2, PDK3, PDK4 and macrophage populations. Building upon these observations, we considered that PDK family genes might yield direct or indirect effects on macrophage polarization, subsequently orchestrating the biological process and clinical outcome of diverse tumors. However, the confirmation of these speculations warrants more comprehensive and robust molecular experiments to solidify our understanding of these potential mechanisms.

This study also has some limitations. Firstly, our findings are constrained by the sequencing technologies and analytical methodologies employed in the database, potentially leading to limitations in data granularity and precision. This common challenge is pervasive in the field of bioinformatics research. Secondly, the potential of PDKs as prognostic biomarkers and their applicability in immunotherapy necessitate validation in a larger and more diverse set of clinical samples. Lastly, acknowledging the inherent constraints of bioinformatics analyses, our intention is to explore and verify the potential mechanisms through several molecular and animal experiments in the near future.

In summary, our study has illuminated the intricate and multifaceted roles of PDK family members expression in driving cancer progression and influencing clinical outcome. These findings need further investigation, explaining the potential of PDKs as promising prognostic biomarkers, particularly within specific tumors. Moreover, our research has unveiled the relationship between immune infiltration and distinct members of the PDK family across various cancers, offering valuable insights that could guide the development of novel immunotherapeutic strategy.

### Supplementary Information

Supplementary Figures.

## Data Availability

The data which support the findings of our study are openly available from the TCGA, cBioPortal, UALCAN, WebGestalt and GeneMANIA databases.
