# Supplementary material for: Pan-cancer analysis reveals PDK family as potential indicators related to prognosis and immune infiltration
Source: Sci Rep. 2024 Mar 7;14:5665. doi: 10.1038/s41598-024-55455-1 (PMC10920909; doi:10.1038/s41598-024-55455-1)
Supplement: Supplementary file 1 — Supplementary Figures. [file 41598_2024_55455_MOESM1_ESM.docx]

**Supplementary Material**

**FIGURE S1**

**
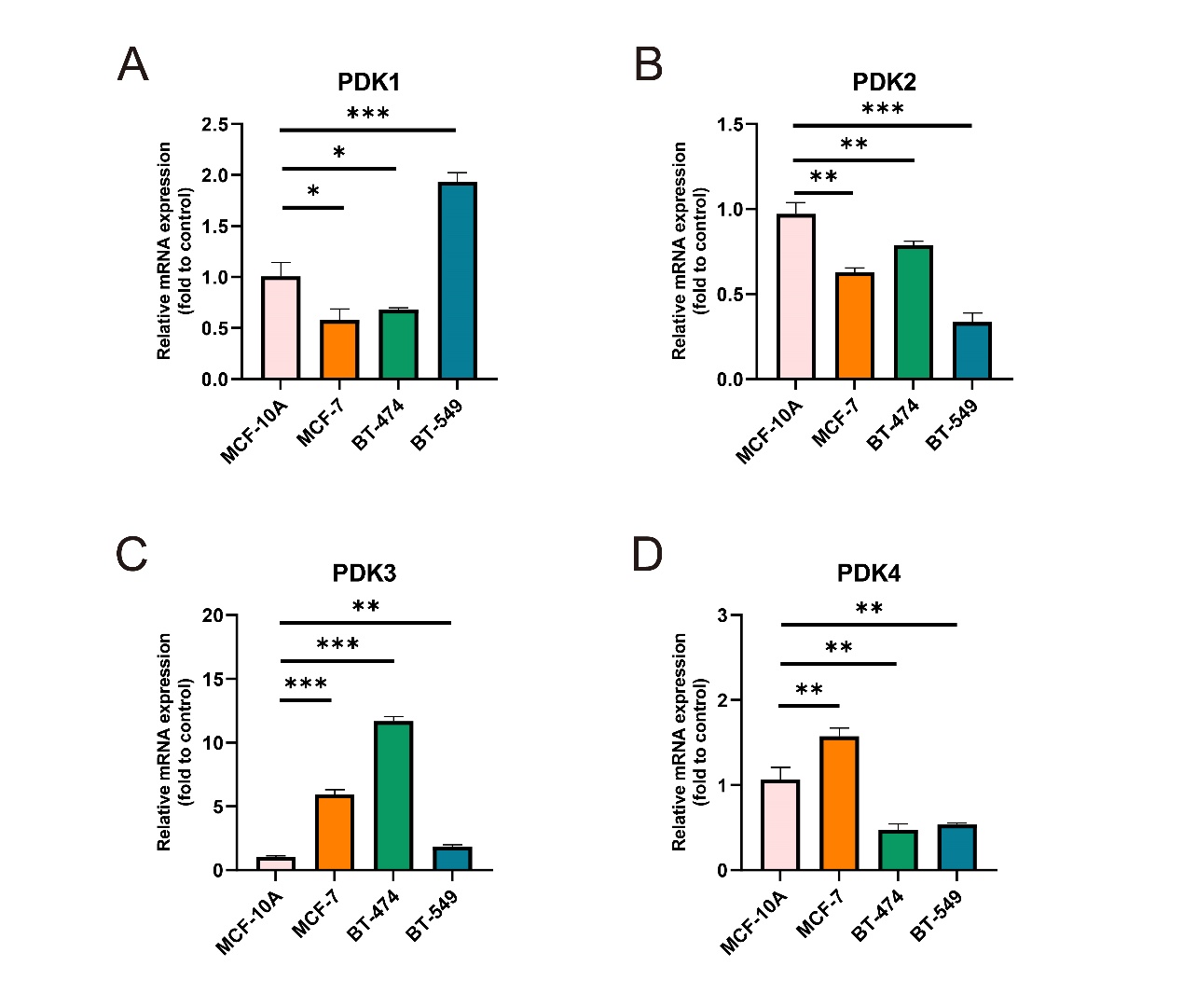
**

**FIGURE S2**

**
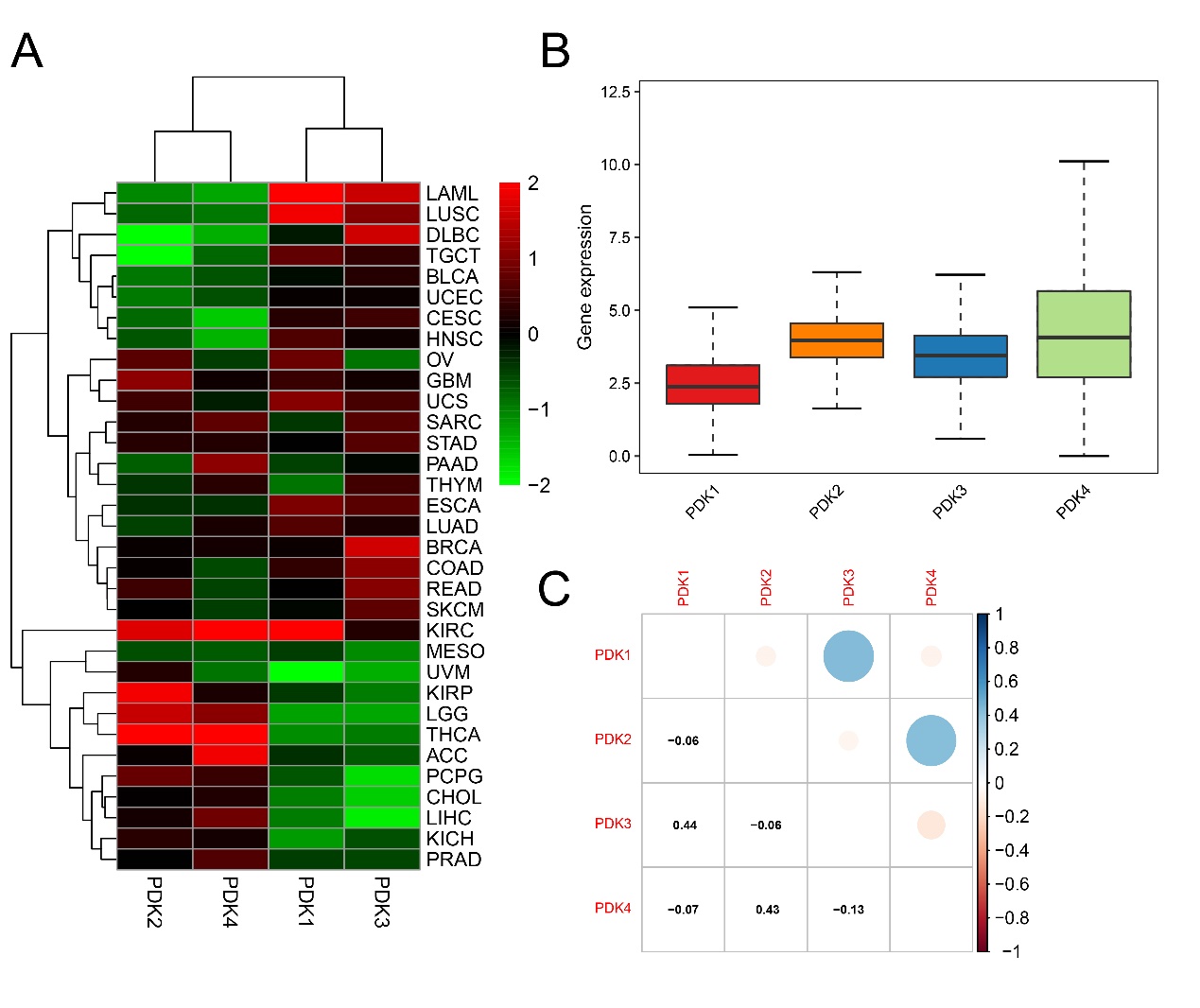
**

**FIGURE S3
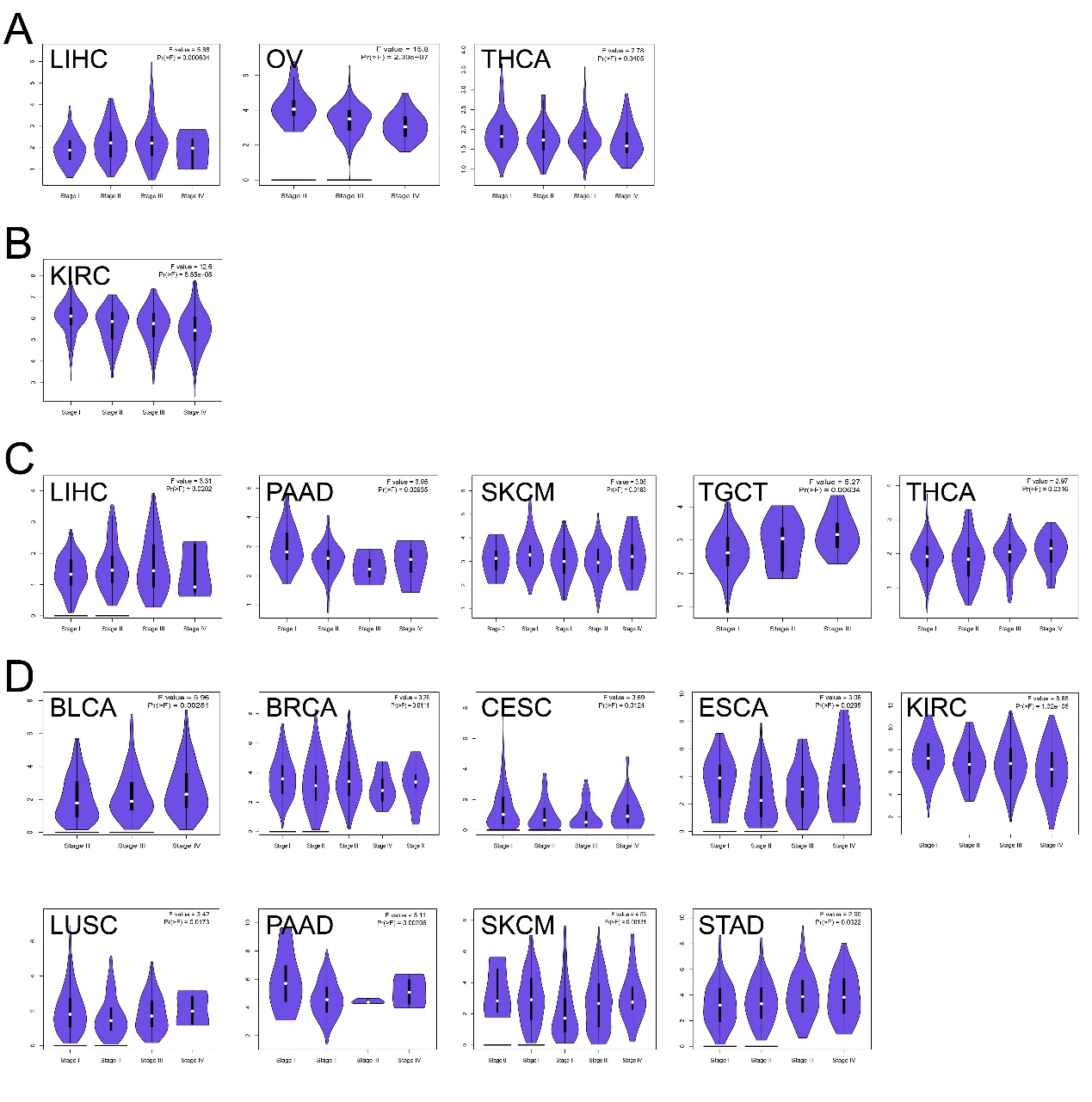
**

**FIGURE S4**

**
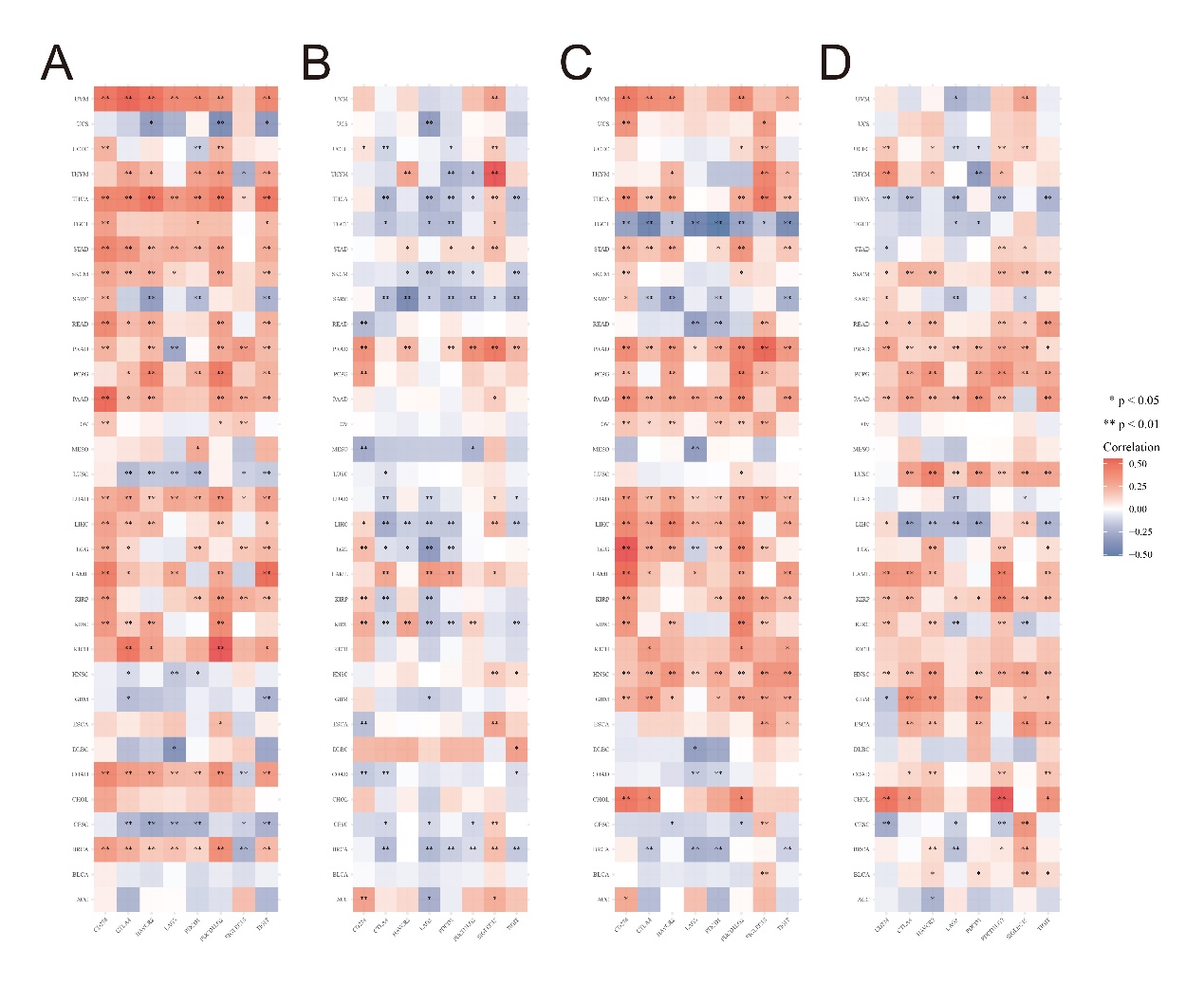
**

**FIGURE S5**

**
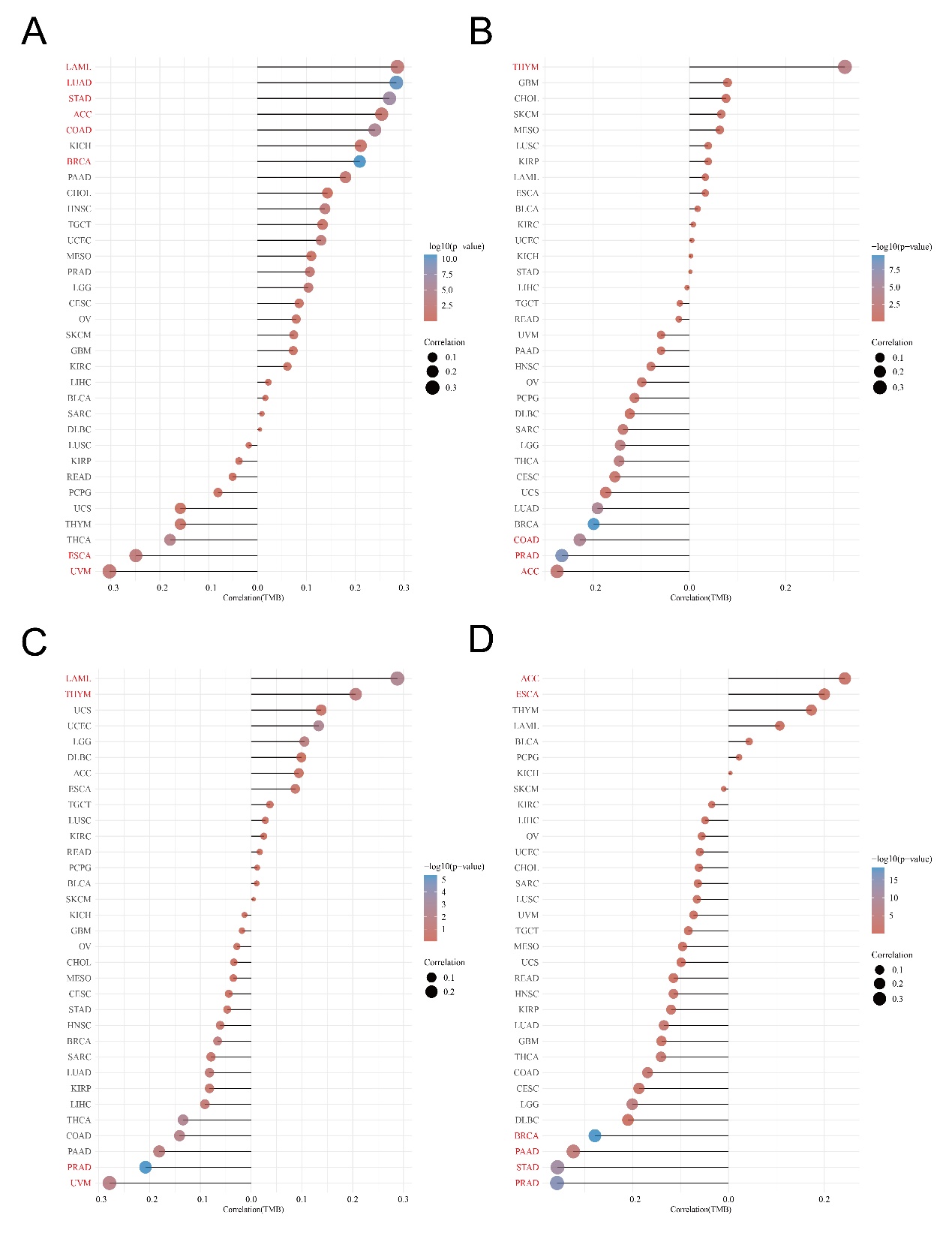
**

**FIGURE S6**

**
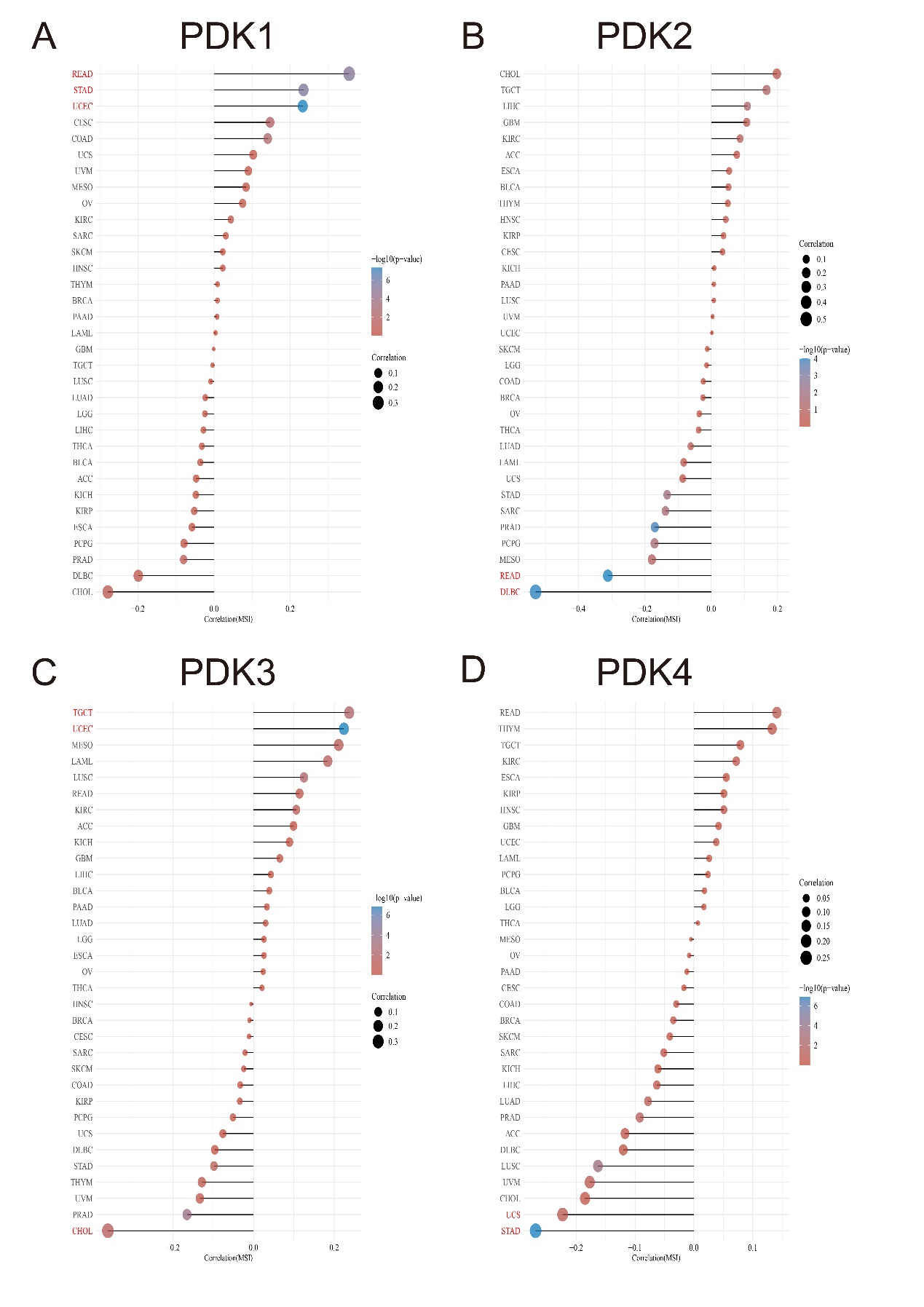
**

**FIGURE S1**

PDKs mRNA expression levels in breast cancer cell lines and human normal mammary epithelial cell quantified by real-time PCR. (A) PDK1, (B) PDK2, (C) PDK3, (D) PDK4. The experiments were repeated three times. *p < 0.05, **p < 0.01, ***p < 0.001.

**FIGURE S2**

PDKs differential expression in pan cancer and correlation analysis between PDKs expression. (A) The heatmap of PDKs differential expression in pan cancer. (B) Boxplot of PDKs expression in 33 TCGA cancer samples. (C) The correlation analysis of PDKs expression. The blue and red dots indicate that PDKs expression level had a negative and positive correlation, respectively.

**FIGURE S3**

Stage-dependent expression level of PDKs in different cancers. Main pathological stages of these cancers were assessed and compared using TCGA data. The log2(TPM + 1) for log-scale was used.

**FIGURE S4**

The correlation analysis between main immune checkpoint members and expression of (A) PDK1, (B) PDK2, (C) PDK3, (D) PDK4. *p < 0.05, **p < 0.01.

**FIGURE S5**

The relationship between TMB and expression of (A) PDK1, (B) PDK2, (C) PDK3, (D) PDK4 in different malignances.

**FIGURE S6**

The relationship between MSI and expression of (A) PDK1, (B) PDK2, (C) PDK3, (D) PDK4 in different cancers.
